# Transmissibility of pandemic H1N1 and genetically related swine influenza viruses in ferrets

**Published:** 2011-01-10

**Authors:** H-L Yen, H Forrest, P Cheung, D Wong, O Li, S Krauss, A Ferguson, JC Crumpton, J Jones, T Choy, E Ma, LLM Poon, GJ Smith, J Nicholls, Y Guan, RG Webster, R Webby, JSM Peiris

**Affiliations:** 1 Department of Microbiology, The University of Hong Kong, Hong Kong SAR; 2 HKU-Pasteur Research Center, Hong Kong, Hong Kong SAR; 3 Department of Pathology, The University of Hong Kong, Hong Kong SAR; 4 St. Jude Children’s Research Hospital, Memphis, TN, USA

## 

Zoonotic infections with swine influenza viruses have occurred sporadically in the past. However, sustained human-to-human transmission of a swine virus did not occur until the emergence of the 2009 pandemic H1N1 (H1N1pdm) virus. H1N1pdm possesses a unique gene combination with gene segments derived from the North America triple reassortant (PB2, PB1, PA, HA, NP, and NS) and the Eurasian avian-like (NA and M) swine influenza viruses.

To identify molecular determinants that enable sustained human-to-human transmission, we compared the direct contact and aerosol transmission efficiency of the pandemic viruses with related swine influenza viruses in ferrets. The transmission potential of seasonal H3N2 [A/Wuhan/359/95 (Wuhan95)], H1N1pdm [A/California/4/09 (CA04) and A/HK/415742/09 (HK415742)], and genetically related swine influenza viruses [A/sw/HK/4167/99 (H1N1) (swHK4167), A/sw/Arkansas/2976/ 02 (H1N2) (swAR2976), A/sw/HK/915/04 (H1N2) (swHK915) and A/sw/HK/201/ 10 (H1N1) (swHK201)] were studied. Ferrets were inoculated with 10^5^ TCID_50_ of the virus. Naïve direct contact and aerosol contact ferrets were introduced at 1day post-inoculation (dpi). Transmission was defined by detection of virus from nasal washes and/ or seroconversion.

We observed direct contact transmission from inoculated donor ferrets to their cage-mates was observed for all viruses studied, albeit at different efficiency. Classical swine-like swHK4167 showed least efficient contact transmission as virus could be detected from all (3/3) direct contacts only at 6 dpi while viral shedding was detected at 4 dpi in other direct contact groups. Aerosol transmission was detected with human seasonal influenza virus Wuhan95 (2/3), H1N1pdm influenza virus CA04 (3/3), HK415742 (2/3), and swine precursor virus swHK915 (1/3). Transmission of Wuhan95 or CA04 to aerosol contacts was detected at 4 or 6 dpi, while transmission of swHK915 was detected later at 8 dpi. While the swine influenza viruses studied were able to transmit via the direct contact route, only swHK915 which shares a common genetic derivation for 7 genes with H1N1pdm possessed capacity for aerosol transmission, albeit of moderate efficiency. SwHK915 differed from swine triple reassortant viruses in the origins of its M gene. It is possible that the M gene derived from Eurasian avian-like swine viruses contributes to the aerosol transmissibility of H1N1pdm influenza viruses.

